# Kiwi Fruits Preservation Using Novel Edible Active Coatings Based on Rich Thymol Halloysite Nanostructures and Chitosan/Polyvinyl Alcohol Gels

**DOI:** 10.3390/gels8120823

**Published:** 2022-12-13

**Authors:** Constantinos E. Salmas, Aris E. Giannakas, Dimitrios Moschovas, Eleni Kollia, Stavros Georgopoulos, Christina Gioti, Areti Leontiou, Apostolos Avgeropoulos, Anna Kopsacheili, Learda Avdylaj, Charalampos Proestos

**Affiliations:** 1 Department of Material Science and Engineering, University of Ioannina, 45110 Ioannina, Greece; 2 Department of Food Science and Technology, University of Patras, 30100 Agrinio, Greece; 3 Laboratory of Food Chemistry, Department of Chemistry, National and Kapodistrian University of Athens, Zografou, 15771 Athens, Greece

**Keywords:** thyme oil, halloysite, chitosan, polyvinyl alcohol, gel, active coatings, nanostructures, kiwi fruits

## Abstract

The concept of this study is the replacement of previous fossil-based techniques for food packaging and food shelf-life extension, with novel more green processes and materials following the spirit of circular economy and the global trend for environmentally positive fingerprints. A novel adsorption process to produce thymol-halloysite nanohybrids is presented in this work. The high dispersion of this thymol-halloysite nanostructure in chitosan biopolymer is one of the goals of this study. The incorporation of this biodegradable matrix with poly-vinyl-alcohol produced a very promising food-packaging film. Mechanical, water-oxygen barrier, antimicrobial, and antioxidant properties were measured. Transparency levels were also tested using a UV-vis instrument. Moreover, the developed films were tested in-vivo for the preservation and the extension of the shelf-life of kiwi fruits. In all cases, results indicated that the increased fraction of thymol from thyme oil significantly enhances the antimicrobial and antioxidant activity of the prepared chitosan-poly-vinyl- alcohol gel. The use of the halloysite increases the mechanical and water-oxygen barrier properties and leads to a control release process of thymol which extends the preservation and the shelf-life of kiwi fruits. Finally, the results indicated that the halloysite improves the properties of the chitosan/poly-vinyl-alcohol films, and the thymol makes them further advantageous.

## 1. Introduction

Nowadays, the prevalence of the circular economy spirit requires the replacement of conventional packaging plastics, which are derived from fossil resources, with biopolymers which are produced via the valorization of food and/or agricultural by-products [1,2]. Moreover, the greenhouse effect imposes the turn to more environmentally friendly activities in all life sectors. Finally, the food shortage and rising prices could be handled via the extension of food shelf-life. Under this spirit, this study aimed at the valorization of some food byproducts and the use of natural biodegradable raw materials to improve the preservation of foods. Some of the most frequently used and promising biopolymers for packaging applications are cellulose, starch, gelatin, and chitosan [3,4,5,6,7]. Chitosan is produced by the deacetylation reaction of chitin. Chitin can be extracted via chemical or biotechnological processes from seafood waste such as shrimp, lobster, and crayfish shells [7,8,9,10]. Chitosan has been extensively studied as a promising biopolymer to be used in active packaging films, coatings, and other industrial applications due to its antioxidant and antimicrobial properties [11,12,13,14,15,16]. Its poor mechanical properties can be enhanced by blending with other polymers [17,18] biopolymers [19,20,21] and/or incorporating nano-reinforcements such as nanoclays to give promising chitosan-based biopolymeric nanocomposite gels ready for film preparation [22]. Due to CS’s water swelling, it has lower water barrier properties than other packaging materials but is well known for its good gas barrier properties [23].

Moreover, modern food packaging technologies follow the incorporation of natural preservatives, antioxidants, and antimicrobials such as essential oils (EOs) into the polymer matrix of the biopolymer, targeting the development of gels exhibiting controlled release properties of EOs into the food, and sequentially to the increase of food self-life and food safety [24,25,26,27,28,29]. Various procedures have been developed to protect the antioxidant and antimicrobial activity of EOs. One of them suggests the encapsulation of EOs into microemulsion or nanoemulsion nanostructures [30,31,32]. Another one suggests the adsorption of such EOs into cheap and naturally abundant adsorbents such as nanoclays and zeolites [33,34,35,36]. The excellent gas barrier properties combined with the intermediate moisture barrier can reduce fruits respiration rate without interrupting the supply of moisture on them. This makes CS a promising coating material to extend the shelf-life of fruits [37].

The kiwifruit is cultivated in many places in Greece. However, Pieria remains the main area of production [38]. Kiwifruit is unique because of its high nutritional content [39]. In our days there is a major effort from researchers and farmers to find low cost ways to produce, keep in storage, and deliver onto the market, fruits of high quality [38].

During our previous work, we developed a chitosan/polyvinyl alcohol (CS/PVOH) gel which led to composite films and exhibited improved mechanical, gas barrier, and antimicrobial properties compared to the relevant properties of pure CS film [18,40]. Furthermore, we developed a procedure for the adsorption of EOs such as thyme, oregano, and basil oil in montmorillonite and organophilic montmorillonite. The incorporation of these nanohybrids in Low-Density Poly-Ethylene (LDPE), Polystyrene (PS), and Poly-Lactide-Acid (PLA) active packaging films was also studied previously [27,36,41,42,43]. A modified method for the adsorption of a fraction rich in thymol from thyme oil (TO) in halloysite nanotube nanoclay (HNT) was applied in this work. The obtained TO@HNT nanohybrids dispersed at 5, 10, and 15 wt.%. nominal content into CS/PVOH matrix via a solution blending method. Pure HNT was also dispersed into CS/PVOH matrix at the same nominal contents and used as reference material. The obtained CS/PVOH/HNT and CS/PVOH/TO@HNT films were characterized via XRD analysis, FTIR spectroscopy, and SEM microscopy. They were also tested for their tensile properties, water/oxygen barrier properties, antioxidant activity, and antimicrobial capacity against food pathogens. Finally, the most active films are applied as a coating to enhance the preservation of kiwi fruits.

## 2. Results and Discussion

The significance of the result of this work could be summarized as the development of a novel active food packaging film using natural raw materials and food industry byproducts and avoiding the use of chemicals and fossil fuel-originated polymers. Two specific goals were achieved in this study, the first is the greener packaging film development and the second is the extension of kiwi fruit shelf-life and beyond this the improved food preservation using such packaging films.

### 2.1. Physicochemical Structural Characterization of TO@HNT Hybrid Nanostructure

**Figure 1:**
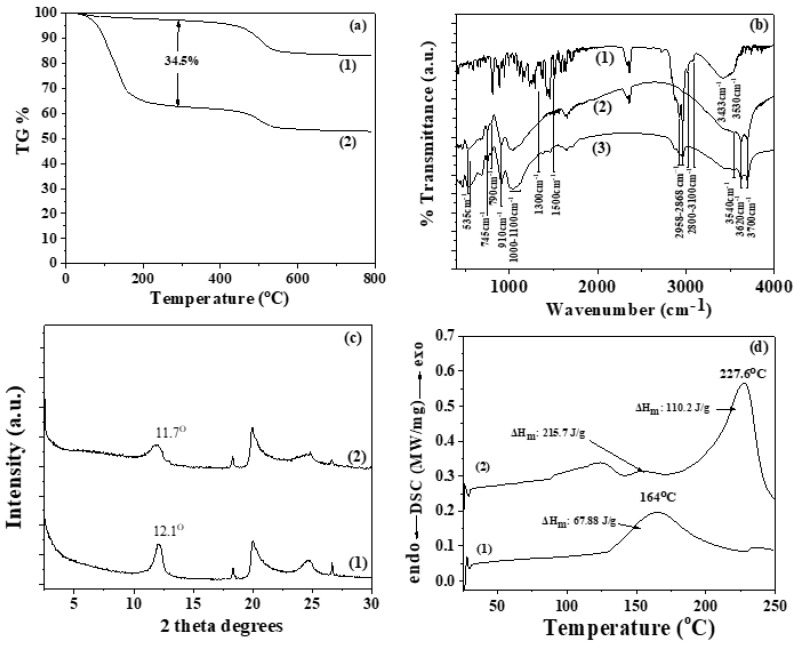
(**a**) TG plots of (1) pure HNT and (2) TO@HNT hybrid nanostructure, (**b**) FTIR plots of (1) TO, (2) pure HNT, and (3) TO@HNT hybrid nanostructure, (**c**) XRD plots of (1) pure HNT, and TO@HNT hybrid nanostructure, and (**d**) DSC plots of pure HNT (line 1) and modified TO@HNT (line 2).

The obtained TO@HNT hybrid nanostructure as well as the pure HNT were characterized with TG experiments, FTIR spectra, XRD analysis, and DSC measurements. TG, FTIR, XRD, and DSC plots of pure HNT and TO@HNT hybrid are shown in Figure 1a–d respectively. TGA plots of both materials in Figure 1a indicate that in both cases exist two mass loss steps. The first one starts at around 50 °C and ends at around 400 °C. The second mass loss step which is attributed to the HNT dehydration process starts at around 450 °C and ends at around 600–700 °C [44,45,46]. In the case of pure HNT, the first mass loss step represents the mass loss of superficially adsorbed water and the second step represents the mass loss of structural/trapped water. In the case of the TO@HNT hybrid, the first step represents the loss of both water and TO molecules. Hence, by subtracting the mass lost from the first mass loss step of pure HNT from the mass lost from the first mass loss step of the TO@HNT hybrid, we calculated an average TO loading on HNT equal to 34.5 wt.%.

In Figure 1b the FTIR spectra of both pure HNT and modified TO@HNT hybrid nanostructure are plotted. In the FTIR plot of TO, a broad peak in the range of 3530 to 3433 cm^−1^ was assigned to the stretching vibration of O-H groups [45]. The bands at ~3100–3000 cm^−1^ are corresponded to aromatic and alkenic -CH=CH- stretch vibrations [36]. The absorption bands in the range 2958 to 2868 cm^−1^ are assigned to the stretching mode of C-H groups [45]. The bands between 1500 cm^−1^ and 1300 cm^−1^ are assigned to the C-H bending of the C-O-H and aliphatic CH_2_ groups bending [36].

In the FTIR plot of pure HNT, the bands at 3700 and 3620 cm^−1^ are assigned to hydroxyl groups in the internal HNT’s surface. The weak band at 3540 cm^−1^ is assigned to the Si–O–Si (Al) groups. The intense absorption bands in the region of 1100–1000 cm^−1^ and at 790 cm^−1^ are assigned to Si–O group. The band at 910 cm^−1^ is assigned to the hydroxyl groups bending vibration. The band at 745 cm ^−1^ is assigned to the Si–O–Al bonds [44,45]. In the FTIR plot of hybrid TO@HNT are assigned the same bands with pure HNT and additionally the characteristic bands of TO mentioned hereabove. The characteristic bands of TO in the FTIR plot of TO@HNT imply the adsorption of TO on the HNT surface. No shift peak of HNT bands was obtained in the FTIR plot of the TO@HNT hybrid implying rather physisorbed than chemisorbed adsorption of TO on the HNT surface.

In Figure 1c the XRD plot of pure HNT and modified TO@HNT powders are shown. In both XRD plots which were obtained, the halloysite’s distinct diffraction peaks at 2θ = 12.0, 20.1, and 24.6 are obvious, and correspond to (001), (100), and (002) planes respectively due to the crystalline property of the HNT [47]. In the case of pure HNT, the presence of the (001) peak at 2θ of 12.1° corresponds to a layer spacing of 0.73 nm. In the case of modified TO@HNT hybrid nanostructure, the peak at 2θ of 11.7◦ corresponds to a layer spacing of 0.76 nm. This difference of approx. 0.03 nm is too small and indicates probably the insertion of small water molecules in HNT’s interlayer space. In the case of thymol molecules insertion in the HNT’s interlayer space, it should be expected a larger opening of HNT’s interlayer space as the thymol molecule size is bigger than that of phenol size (0.4 nm) [36]. So, XRD results indicated that adsorption of thymol took place on the external surface of HNT and no changes in the crystal structure of HNT are obtained due to the TO adsorption process. This result is in accordance with Shemesh et al. [48] where carvacrol a molecule similar to thymol loaded on the external surface of HNT.

In Figure 1d the DSC plots of pure HNT (line (1)) and modified TO@HNT (line (2)), nanohybrid are presented. In the DSC plot of pure HNT, the exothermic peak at 164 °C with a ΔH equal to 67.88 J/g is assigned to the desorption process of water molecules. In the DSC plot of TO@HNT nanohybrid, the exothermic peak at 227 °C with a ΔH equal to 227.6 J/g is assigned to the desorption of TO molecules in accordance with the previous report [49]. Thus, DSC analysis indicates the absorption of rich TO molecules on the HNT surface.

The overall characterization of TO@HNT nanohybrids concludes that a rich in TO fraction is physiosorbed on the external HNT’s surface and validates the distillation/evaporation adsorption process followed.

### 2.2. XRD Analysis of CS/PVOH/HNT and CS/PVOH/TO@HNT Films

**Figure 2:**
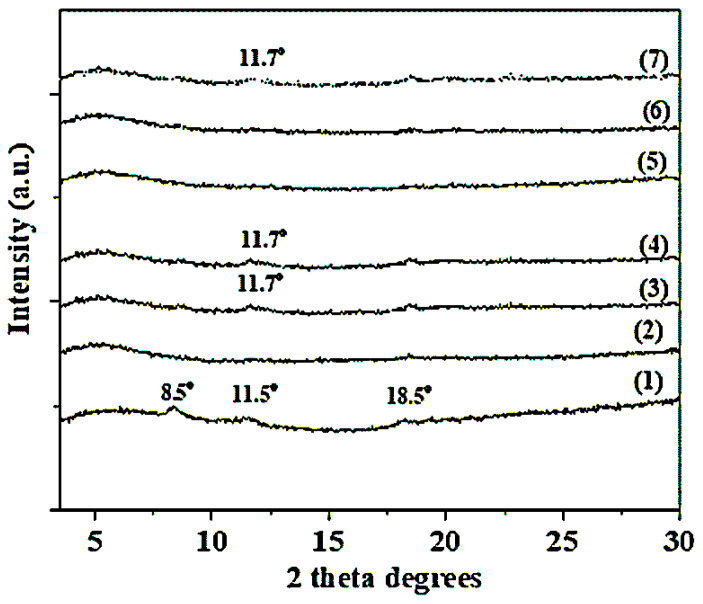
XRD plots of (1) CS/PVOH film, (2) CS/PVOH/5HNT, (3) CS/PVOH/10HNT, (4) CS/PVOH/15HNT, (5) CS/PVOH/5TO@HNT, (6) CS/PVOH/10TO@HNT, and (7) CS/PVOH/15TO@HNT nanocomposite films.

XRD patterns of all obtained CS/PVOH/HNT and CS/PVOH/TO@HNT films as well as of films from pure CS/PVOH gels are shown in Figure 2. The pattern of films from pure CS/PVOH gels exhibits three broad peaks at 8.5°, 11.5°, 18.5°, and 23° are observed. The peaks at 8.5° and 11.4° indicate the CS’s hydrated crystallite structure due to the insertion of water molecules in the CS’s crystal lattice [50,51] while the third peak at 18.5° is assigned to the CS’s regular crystal lattice [51,52]. The later broaden peak around 23° assigned to the amorphous structure of CS [51,52].

In the case of CS/PVOH/xTO@HNT film patterns, the diffraction peaks of HNT at 2θ = 11.7° do not observe for x = 5% wt. and 10% wt., i.e., line (5) and (6), and observed slightly for x = 15%wt., i.e., line (7). In the cases of CS/PVOH/xHNT, the HNT peak does not observe for x = 5%wt., i.e., line (2), and is observed slightly for x = 10%wt. and 15%wt. i.e., lines (3) and (4). These results indicate that in cases of TO@HNT the optimum dispersion of nanohybrid achieved for concentration x = 10%wt., while for concentration x = 15%wt. the aggregation started. In cases of HNT the optimum dispersion of nanohybrid achieved for concentration x = 5%wt. while for concentration x = 10%wt. or greater the aggregation started. The absence of shift of basal HNT’s peak at 2θ = 11.7° indicates that CS/PVOH chains can not intercalate HNT’s interlayer space [53]. The absence of HNT’s peak in all CS/PVOH/TO@HNT films implied the higher dispersity of modified TO@HNT hybrid nanostructure than pure HNT in the CS matrix. The high dispersion of HNT and TO@HNT in the CS matrix is beneficial for such nanocomposite films.

### 2.3. FTIR Spectroscopy of CS/PVOH/HNT and CS/PVOH/TO@HNT Films

**Figure 3:**
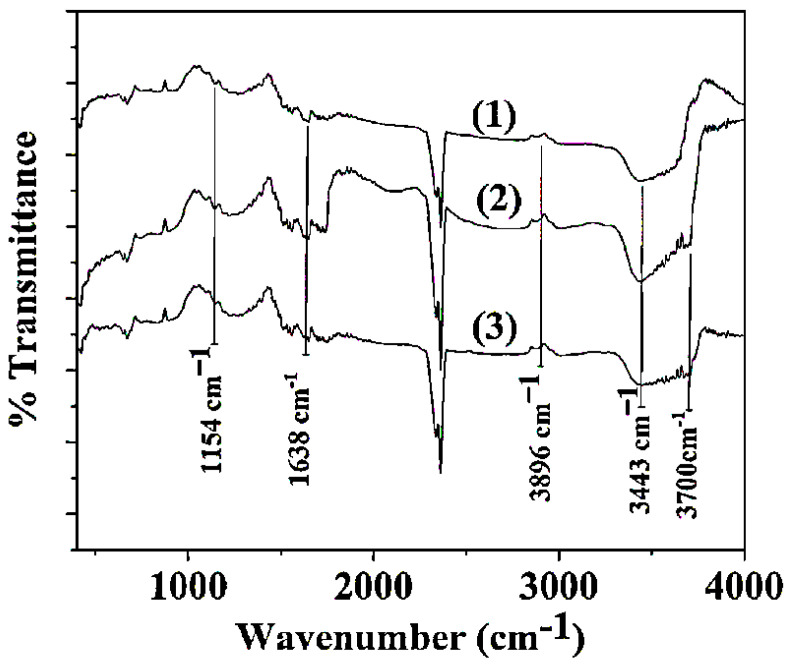
FTIR spectras of neat CS/PVOH film (1) as well as CS/PVOH/HNT (2), and CS/PVOH/TO@HNT (3) films for comparison.

In Figure 3 representative spectra of pure CS/PVOH, CS/PVOH/HNT, and CS/PVOH/TO@HNT are observed.

FTIR spectra of pure CS/PVOH (see line (1) in Figure 3) are a combination of both CS and PVOH reflections. The large band at 3443 cm^−1^ is assigned to O–H groups stretching both presented in CS and PVOH and to N–H groups stretching of CS. The same band is also assigned to the intra- and inter-molecular hydrogen bonds of the CS/PVOH matrix.Τhe band at2896 cm^−1^ is assigned to C–H asymmetric and symmetric stretching from CH_2_ and CH groups. The band at 1637–1644 cm^−1^ is assigned to the associated water, C–OH from the glycosidic units of CS chains, and also the presence of residual N-acetyl groups (C=O stretching of amide I), and N-H in-plane deformation coupled with C–N stretching of amide II (secondary amide) from CS. The band at 1154 cm^−1^ is assigned to the glycosidic linkage (asymmetric bridge stretch) [54].

In the FTIR spectra of CS/PVOH/HNT and CS/PVOH/TO@HNT systems (see lines (2) and (3) in Figure 3) it additionally obtained the characteristic reflection band at 3620 cm^−1^ assigned to O-H groups in the internal HNT’s surface. With a careful glance, it is obtained that the main difference between FTIR plots of CS/PVOH/HNT and CS/PVOH/TO@HNT is that in the case of CS/PVOH/HNT plot the band of O-H and N-H group stretching at 3443 cm^−1^ is more intense than the same band of CS/PVOH/TO@HNT plot. This implies a higher interaction between OH groups of the CS/PVOH matrix and OH groups of pure HNT than OH groups of the CS/PVOH matrix and modified TO@HNT.

### 2.4. SEM Images of CS/PVOH/HNT and CS/PVOH/TO@HNT Films

A Scanning Electron Microscopy (SEM) instrument was used for the surface/cross-section morphology investigation of the pure CS/PVOH film as well as of the CS/PVOH/HNT and CS/PVOH/TO@HNT nanocomposite films. The results confirmed that both HNT and the TO@HNT hybrid nanostructures were homogeneously dispersed in the polymer matrix. The chemical elements of the pure and the final nanocomposite active packaging films were identified by carrying out an Energy dispersive spectrometer (EDS) analysis on the surface of the materials.

**Figure 4:**
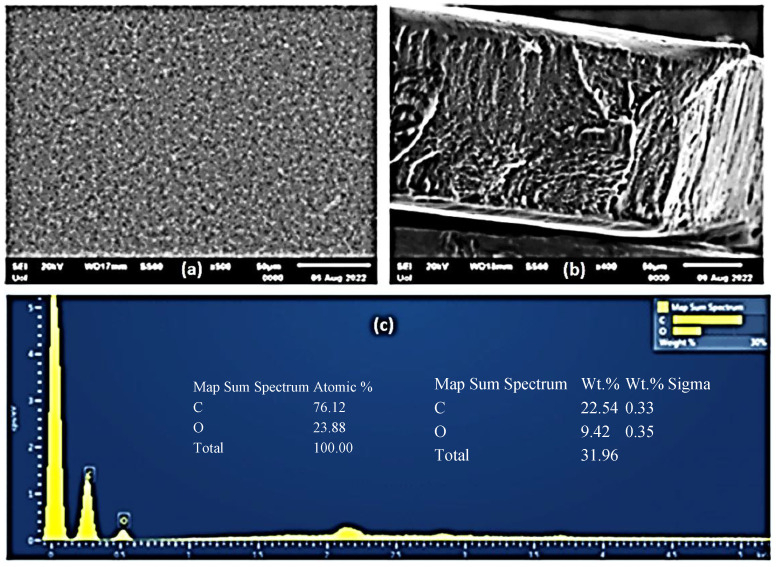
(**a**) SEM images of the surface and (**b**) cross-section for the pure CS/PVOH film. (**c**) Energy dispersive spectrometer (EDS) spectrum and relative elemental analysis of the surface (inset) from the SEM image (**a**).

The SEM images in Figure 4a,b show the expected smooth morphology of the neat polymer. The EDS spectra in Figure 4c certify the existence of carbon (C), and oxygen (O).

**Figure 5:**
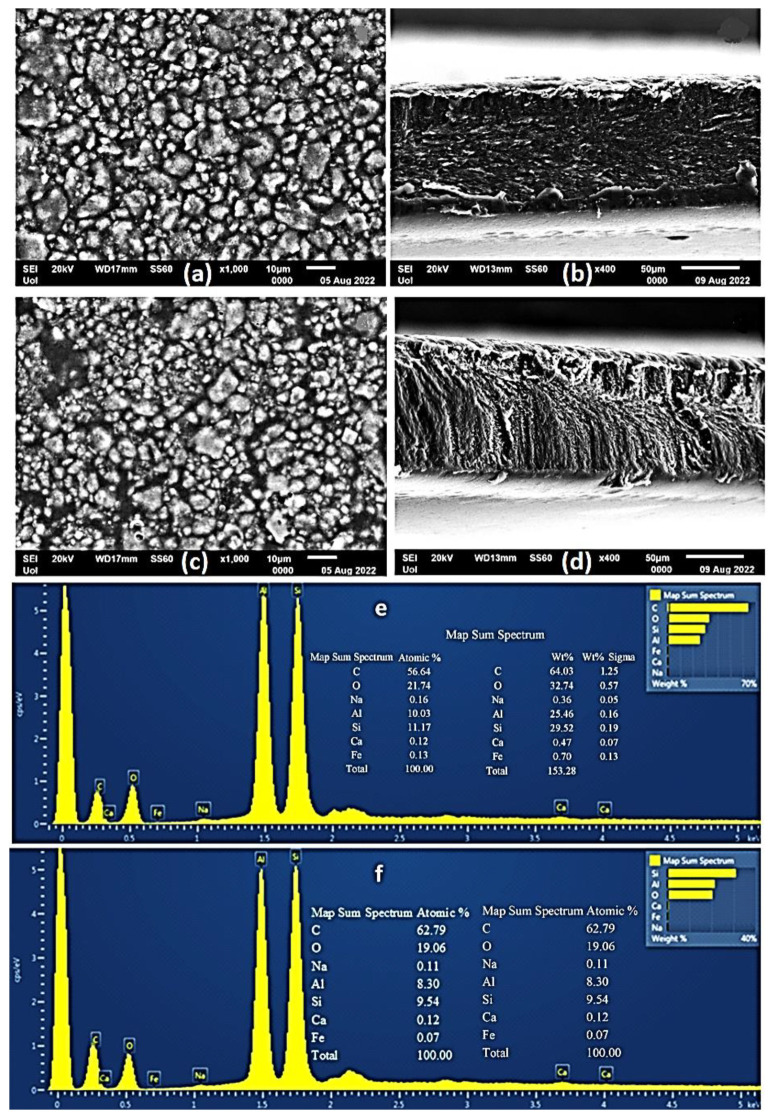
(**a**,**c**) SEM images of the surface and (**b**,**d**) cross-section for the nanocomposite films of ALG/G/5HNTNZ (**a**,**b**) and ALG/G/5TO@HNT (**c**,**d**) respectively. (**e**,**f**) Energy dispersive spectrometer (EDS) spectrum and relative elemental analysis of the surface (inset) from the SEM images (**a**,**c**).

**Figure 6:**
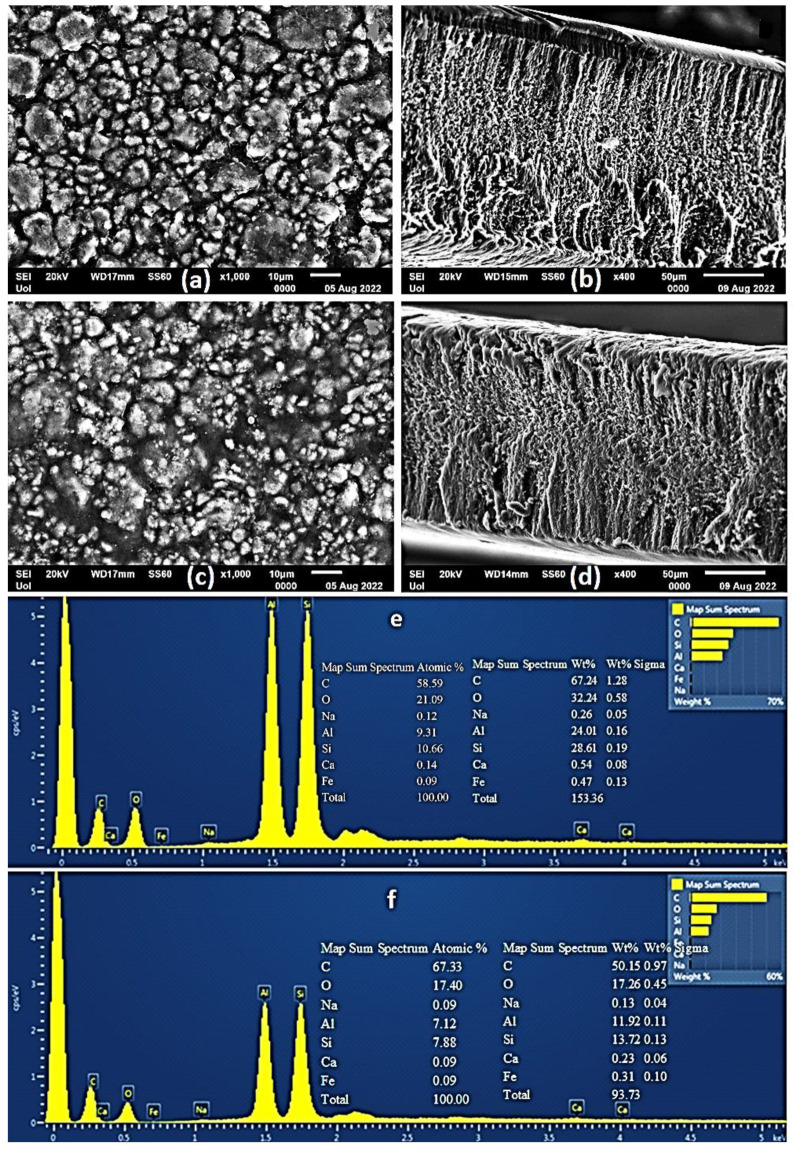
(**a**,**c**) SEM images of the surface and (**b**,**d**) cross-section for the nanocomposite films of ALG/G/10HNT (**a**,**b**) and ALG/G/10TO@HNT (**c**,**d**) respectively. (**e**,**f**) Energy dispersive spectrometer (EDS) spectrum and relative elemental analysis of the surface (inset) from the SEM images (**a**,**c**).

**Figure 7:**
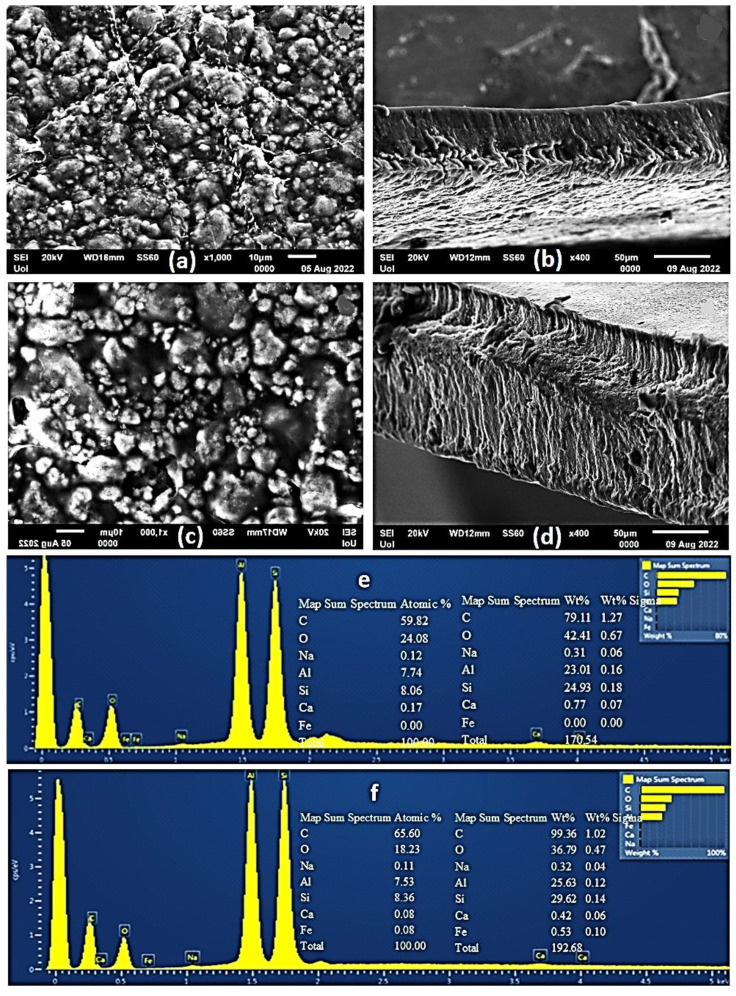
(**a**,**c**) SEM images of the surface and (**b**,**d**) cross-section for the nanocomposite films of ALG/G/15HNT (**a**,**b**) and ALG/G/15TO@HNT (**c**,**d**) respectively. (**e**,**f**) Energy dispersive spectrometer (EDS) spectrum and relative elemental analysis of the surface (inset) from the SEM images (**a**,**c**).

Surface and relative cross-section images of CS/PVOH/HNT and CS/PVOH/TO@HNT with different ratios of HNT and TO@HNT are presented in Figure 5, Figure 6 and Figure 7. Figure 5e,f, Figure 6e,f and Figure 7e,f show EDS chemical analysis of nanocomposite active packaging films with different concentrations of pure HNT and TO@HNT hybrid nanostructure i.e., 5, 10, and 15 wt.%. The presence of typical elements such as Si, Al, O, Fe, and Ca was confirmed.

As illustrated in Figure 5, Figure 6 and Figure 7, the content of both HNT and TO@HNT increases the aggregation of the obtained CS/PVOH/HNT, and CS/PVOH/TO@HNT nanocomposites decrease further. Nevertheless, SEM images of the final nanocomposite films show that both pure HNT and TO@HNT nanohybrid were homogeneously dispersed and suggest enhanced compatibility with the polymer matrix. Moreover, SEM surface and cross-section images were shown more homogenous dispersion in the case of TO@HNT hybrid nanostructure in nanocomposite films compared to the relevant of pure HNT. This means that the TO@HNT hybrid nanostructure was incorporated significantly better in the polymer matrix compared to the incorporation of the respective pure HNT.

### 2.5. Tensile Properties of CS/PVOH/HNT and CS/PVOH/TO@HNT Films

**Figure 8:**
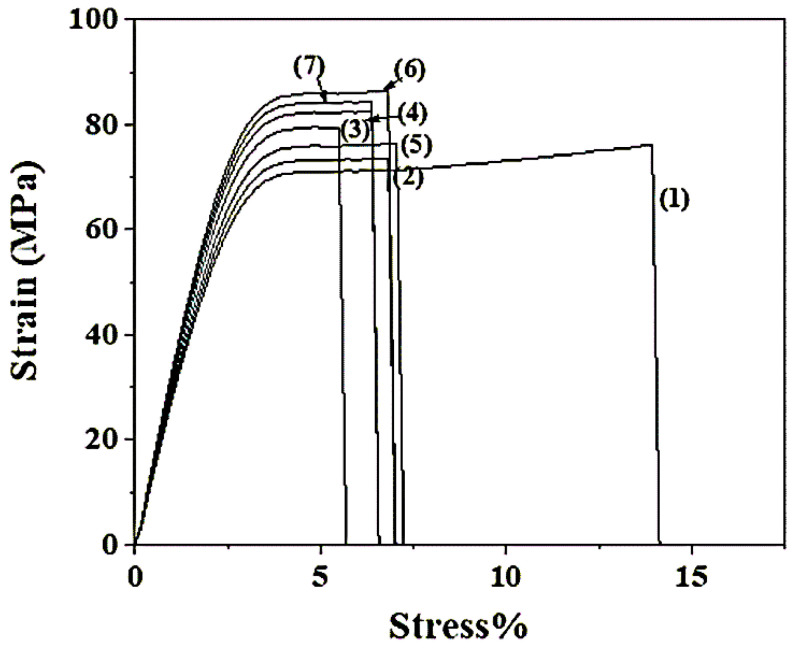
Stress-strain curves of (1) CS/PVOH, (2) CS/PVOH/5HNT, (3) CS/PVOH/10HNT, (4) CS/PVOH/15HNT, (5) CS/PVOH/5TO@HNT, (6) CS/PVOH/10TO@HNT and (7) CS/PVOH/15TO@HNT.

Typical stress-strain curves of all CS/PVOH/HNT and CS/PVOH/TO@HNT films are shown in Figure 8.

From stress-strain curves in Figure 8 the Young’s (E) Modulus, ultimate tensile strength (σ_uts_), and % strain at break (ε_b_) values were calculated and are listed in Table 1 for comparison.

From the Young’s (E) Modulus, ultimate tensile strength (σ_uts_), and % strain at break (ε_b_) values, which are presented in Table 1, we could assume that CS/PVOH/HNT and CS/PVOH/TO@HNT nanocomposite films are stronger than pure CS/PVOH film. The higher the nominal content of the HNT and TO@HNT the higher ultimate strength and lower elongation at break values. In advance, TO@HNT-based nanocomposite films exhibited higher strength values than HNT-based nanocomposites. The increase of tensile strength with HNT and TO@HNT is in agreement with previous reports where HNT loaded in CS/PVOH with nominal content 0–5 wt.%. [53]. Here it is reported for the first time that this increment is existed also for higher HNT nominal loading up to 15 wt.%. It is also reported that a higher tensile increment is taking place for TO@HNT loading compared to the relevant HNT loading. This higher increase of tensile strength with TO@HNT addition is in agreement with the higher dispersion of the TO@HNT in the CS/PVOH matrix as was presented before by the XRD and SEM results.

### 2.6. UV-vis Transmittance of CS/PVOH/HNT and CS/PVOH/TO@HNT Films

**Figure 9:**
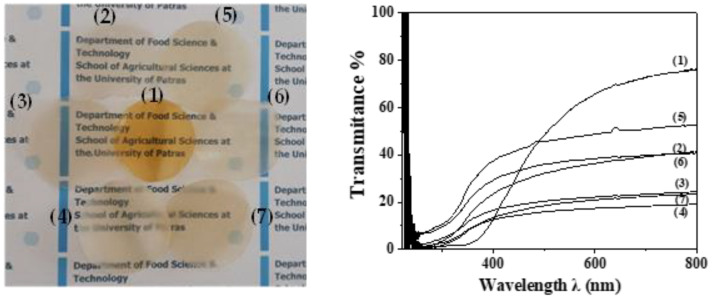
(**a**) photo images and (**b**) UV-vis transmittance plots of (1) pure CS/PVOH films, (2) CS/PVOH/5HNT, (3) CS/PVOH/10HNT, (4) CS/PVOH/15HNT, (5) CS/PVOH/5TO@HNT, (6) CS/PVOH/10TO@HNT, and (7) CS/PVOH/5TO@HNT films.

In Figure 9 photo images (see Figure 9a) and UV-vis plots (see Figure 9b) of all prepared films are shown for comparison.

As it is obtained in UV-vis transmittance plots and illustrated in film images the addition of both HNT and TO@HNT decreases the transmittance of films. From UV-vis transmittance plots is obtained that TO@HNT-based films exhibited higher transmittance than HNT-based films. This result is in accordance with the higher dispersity of TO@HNT hybrid nanostructure in the CS/PVOH matrix which was discussed above in XRD and SEM results.

### 2.7. Water-Oxygen Barrier Properties of CS/PVOH/HNT and CS/PVOH/TO@HNT Films

In Table 2 the water-oxygen transmission rate values for all CS/PVOH/HNT and CS/PVOH/TO@HNT films as well as pure CS/PVOH films are listed. By multiplying water-oxygen transmission rate values with film thickness the water diffusivity (D) and the oxygen diffusivity (Pe_O2_) values are obtained and are listed in Table 2 also for comparison.

For both water and oxygen diffusivity values, in general, could be stated that: (i) the addition of TO@HNT causes a higher increase of water-oxygen barrier than the addition of HNT, (ii) the highest water and oxygen barrier is achieved for films containing 10 wt.%. HNT and 10 wt.%. TO@HNT, and (iii) the film with the optimum water-oxygen barrier is CS/PVOH/10TO@HNT. So, it is concluded that around 10 wt.%. is the optimum content for both HNT and TO@HNT nanostructures to obtain the highest water/oxygen barrier. This conclusion is consistent with the results of the XRD measurements mentioned above herein. In other words, with 10 % wt. addition of both HNT and TO@HNT in the CS/PVOH matrix the optimum dispersions are achieved to obtain the highest water/oxygen barrier.

### 2.8. Antioxidant Activity of CS/PVOH/HNT and CS/PVOH/TO@HNT Films

Antioxidant activity in active food packaging has a key role to extend the shelf-life of food products. Especially for such edible coatings enhances nutritional and aesthetic quality aspects of food without affecting its integrity.

The calculated % antioxidant activity values of all CS/PVOH/HNT and CS/PVOH/TO@HNT films as well as pure CS/PVOH film are obtained in Table 3.

As it is observed no significant antioxidant activity is obtained for pure CS/PVOH and CS/PVOH/HNT films. For CS/PVOH/TO@HNT films antioxidant activity is increased as the TO@HNT nominal content is increased. The highest antioxidant activity value is obtained for CS/PVOH/15TO@HNT film.

### 2.9. Antibacterial Activity of CS/PVOH/HNT and CS/PVOH/TO@HNT Films

**Figure 10:**
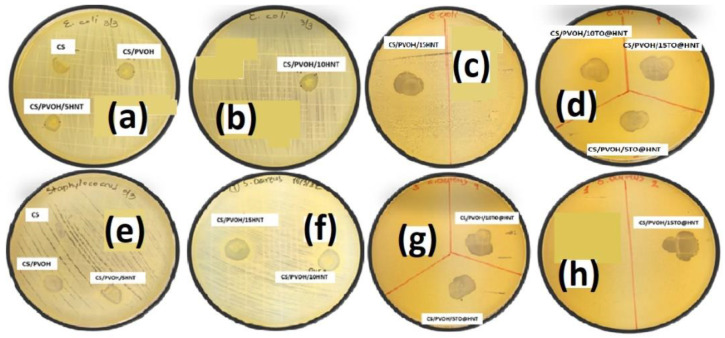
Petri dishes images of (**a**–**c**,**e**,**f**) CS, CS/PVOH, CS/PVOH/xHNT, and (**d**,**g**,**h**) CS/PVOH/xTO@HNT films against *E. coli, S. aureus, S. enterica*, and *L. monocytogenes*.

Figure 10 depicts the petri dishes used for the antimicrobial activity measurements of CS, CS/PVOH, CS/PVOH/xHNT, and CS/PVOH/xTO@HNT films against *E. coli* and *Staphylococcus* bacteria. Table 4 presents the antibacterial activity of the developed films that were based on the CS/PVOH nano-reinforcement. Four foodborne pathogenic bacteria cultivations i.e., *Escherichia coli*, *Staphylococcus aureus*, *Salmonella enterica*, and *Listeria monocytogenes* were used to test the antibacterial capacity of all films. The clear zone’s diameter around the tested films indicates the magnitude of the inhibition of the microorganisms’ growth. The absence of a clear zone, which means zero value of diameter, entails the absence of an inhibitory zone. Moreover, in this work, the bacteria growth in the area of direct contact of film with the agar surface was also studied.

The chitosan films CS/PVOH/5HNT, CS/PVOH/10HNT, CS/PVOH/15HNT, CS/PVOH/5TO@HNT, CS/PVOH/10TO@HNT, CS/PVOH/15TO@HNT were compared to pure CS and CS/PVOH films. Pure CS films inhibited the growth of all tested bacteria but only by direct contact; except *L. monocytogenes* where no antibacterial activity was observed either in the contact area or by the formation of clear surroundings zones.

Furthermore, the CS/PVOH film showed antibacterial activity by formatting a clear zone of 3.50 mm for *E. coli*, 3.83 mm for *S. enterica*, and, 4.47 mm for *S. aureus* while no antibacterial effect against *L. monocytogenes* was observed.

All the incorporated CS films displayed antibacterial effectiveness. The noted inhibition of the bacteria growth seems to have a dependency on the HNT and thyme oil (TO) concentration.

By reviewing the results, it is obvious that the growth inhibition was amplified upon increasing the concentration of the nanostructures and the EO. The CS/PVOH/HNT (5%, 10%, 15%) films showed pronounced antibacterial activity against the tested bacteria.

Specifically, the CS/PVOH/15HNT film exhibited higher antibacterial activity against *E. coli*, *S. enterica*, *S. aureus*, and *L. monocytogenes* if compared to CS/PVOH/5HNT, and CS/PVOH/10HNT. The inhibitory clear zones were noticeably higher for CS/PVOH/HNT films when thyme oil (TO) was incorporated.

The CS/PVOH/5TO@HNT film inhibited all the tested bacteria by formatting clear zones of 7.50 mm for *E. coli*, 7.00 mm for *S. enterica*, 9.00 mm for *S. aureus*, and 8.00 mm for *L. monocytogenes.*

Increasing the thyme oil concentration, it was also enhanced the zone of inhibition of bacteria growth. Finally, CS/PVOH/HNT films containing 15% thyme oil displayed the highest antibacterial activity, resulting in a clear zone formation of 8.00 mm for *E. coli*, 9.00 mm for *S. enterica*, 10.00 mm for *S. aureus*, and 9.00 mm for *L. monocytogenes.*

In all cases, the nano-enforcement films showed significant antibacterial activity against Gram-negative bacteria and slightly stronger activity against Gram-positive bacteria.

It is known that chitosan possesses important antibacterial activity against a wide spectrum of bacteria. This activity is ascribed to its cationic nature (positively charged ammonium (NH_4_^+^)) that interacts with the negatively charged compounds of the bacteria cell wall [55]. However, CS does not show any migrated inhibitory activity [56]. Bacterial cell wall barry a negative charge, therefore electrostatic interaction between bacteria and positively-charged clays such as HNT, under specific conditions (pH, ionic force) is probable [57]. In order for clay to exhibit antibacterial activity, it is crucial to have the ability to maintain metal ions in solution and to have also sufficient interlayer cation exchange capacities [58]. Theoretically, HNTs do not meet these criteria, however, the literature refers to a wide range of possible modes of action of HNTs against bacteria. Abhinayaa et al., 2019 found that HNT at a concentration of 2.5 mg mL^−1^, was able to inhibit the growth of the phytopathogenic bacteria *Agrobacterium tumifaciens* and *Xanthomonas oryzae*, while at lower concentrations it was observed decreased bacteria growth rate and damages on the cell membrane. These results were probably attributed to the effect of siloxane groups of HNTs surface in combination with the production of reactive oxygen species [59]. Moreover, increased antibacterial activity has been observed after the modification of the HNT surface. The functionalized HNT displayed strong antibacterial activity against the food-borne bacteria, *L. monocytogenes*, and *E. coli* [60,61].

HNT has been also found to be toxic to *E. coli* and *Salmonella typhimurium*, especially as a result of light-dependent oxidative stress [59,62]. Several antibacterial compounds such as antibiotics, essential oils, antibacterial peptides, etc. have been loaded into the HNTs in order to enhance the antibacterial activity [63,64]. The HNT structure seems to allow the sustained release of the incorporated antibacterial agents such as thyme oil, leading to bacteria inhibition. By this controlled release, the thyme oil could enter the lipid layer of the bacterial cell wall causing cell death. Moreover, the interaction of bacterial cell walls with the thyme oil-HNT matrix may produce an oxidation-reduction response leading to cell death due to the production of reactive oxygen species [65].

Concluding, it is once more noted that in the present work, the bacteria growth was inhibited in a dose-dependent manner. The antibacterial efficacy of the tested films could be due to the nanocomposite films themselves but also might be due to the controlled release/migration of thyme oil. Many parameters play a role in the final antibacterial effect of a nanostructure, such as bacterial strain, nanoparticle type/size, chitosan molecular weight, growth media type, assay type, and bacterial cell concentration. Consequently, the increased antibacterial activity of the CS/PVOH/HNT films reported in this work is attributed to the synergistic effect of chitosan, HNT, thyme oil concentration, etc.

### 2.10. Packaging Test-Application of CS/PVOH/HNT and CS/PVOH/TO@HNT Films as Coating on Fresh Kiwifruits

**Figure 11:**
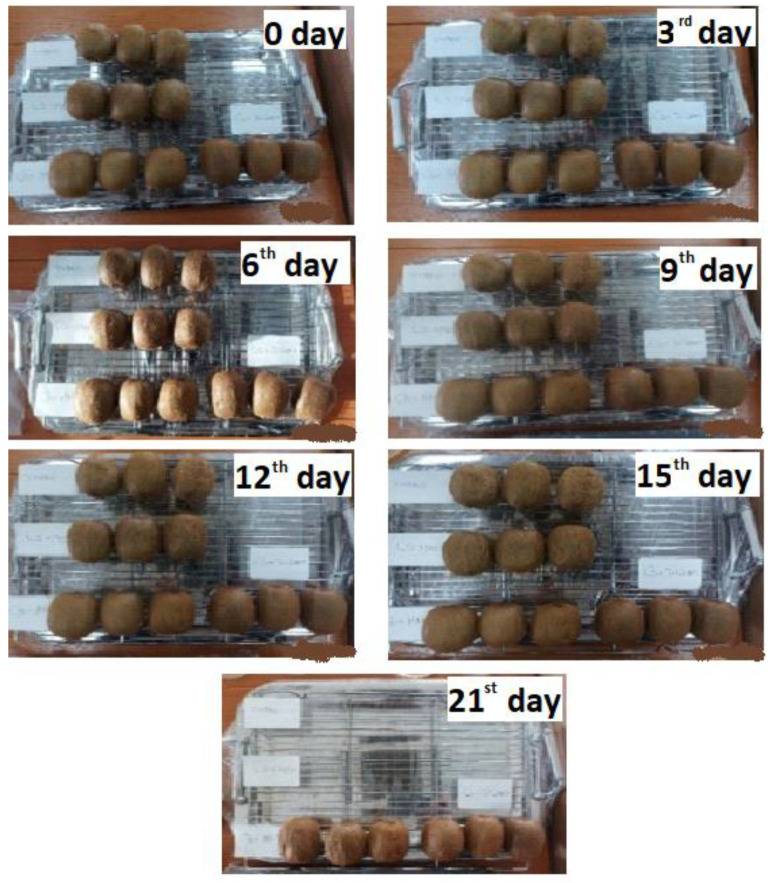
Images of uncoated kiwifruits, coated with CS/PVOH solution kiwifruits, coated with CS/PVOH/10HNT solution kiwifruits and coated with CS/PVOH/10TO@HNT solution kiwifruits stored at 25 °C and ambient humidity at zero, 3rd, 6th, 9th, 12th, 15th and 21st day.

In Figure 11 of uncoated kiwifruits and coated kiwifruits with CS/PVOH, CS/PVOH/10HNT, and CS/PVOH/10TO@HNT solution after 21 days of storage at 25 °C and ambient humidity are shown. As it is obtained from the uncoated kiwifruits the weight loss change is visible from the 3rd day of storage. The visible changes for uncoated samples are more visible on the 6th day and the deterioration increased until the 15th day when the uncoated kiwi fruits were rejected. In the kiwifruits coated with pure CS/PVOH solution the deterioration is visible 3 days later in the 6th day and is increased until the 15th day when also these samples were rejected. For the kiwifruits coated with CS/PVOH/10HNT and CS/PVOH/10TO@HNT solution, the deterioration is much slower. The first visible weight loss change is starting for the kiwifruits coated with CS/PVOH/10HNT solution on the 12th day while for the kiwifruits coated with CS/PVOH/10TO@HNT solution three days later on the 15th day. From the 15th to 21st day of storage as it is obtained (see the last image in the down part of Figure 11) the deterioration of kiwifruits coated with CS/PVOH/10HNT was much more accelerated than the kiwifruits coated with CS/PVOH/10TO@HNT solution which are in a much better optical condition in the last day of the experiment. To conclude a significant deceleration of kiwifruits deterioration was obtained for all coated samples. The uncoated kiwifruits deterioration starts on the 3rd day, while for the kiwifruits coated with CS/PVOH, CS/PVOH/HNT, and CS/PVOH/10TO@HNT solution on the 9th, 12th, and 15th day correspondingly. At this point, it must be mentioned, that the delayed deterioration of the kiwi fruits could be attributed also to the antimicrobial activity of the tested films. Although the films were studied for certain food-borne bacteria, it is likely to have also antimicrobial activity against other spoilage microorganisms, leading to extended life.

## 3. Conclusions

Concluding this study, according to the TG and FTIR results the proposed modified adsorption process led to ~35 wt.%. of TO loading onto HNT nano-clay combined with very good incorporation. Furthermore, the FTIR results indicate a prevailing physisorption mechanism as it is compared to chemisorption. This led to a controlled release mechanism of the TO which was confirmed by the antibacterial tests. We are heading in the same direction if we interpret the XRD results shown, that the thymol was adsorbed on the external surface of the HNT in a high order and thus, such molecules could be easily released. The comparison of the HNT with the TO@HNT nanostructures behaviour via SEM, EDS, and XRD measurements implies that the dispersion of the second one in the CS matrix is higher than the relevance of the first one, which is beneficial for the final developed film. Even though CS/PVOH/15TO@HNT exhibits higher antioxidant and antibacterial activity, the CS/PVOH/10TO@HNT exhibits a higher water-oxygen barrier. According to the XRD measurements, this result originates from dispersion collapse for higher nanohybrid addition. Such results were verified by the in-vivo experiments of kiwi fruits preservation where the uncoated food started to decline on the 3rd day, the coated food with CS/PVOH/15HNT film started to decline on the 12th day, and the coated food with CS/PVOH/15TO@HNT film started to decline at the 15th day. The rejection day of the uncoated kiwi fruits was the 15th day while the rejection of the coated fruits was the 21st day. The final situation of the CS/PVOH/15TO@HNT kiwi samples was better than the relevant of the CS/PVOH/15HNT coated samples. Finally, UV-vis and SEM measurements show that the TO addition is beneficial for the film’s transparency and to the HNT dispersion in the CS matrix.

As a final result of this study, we could say that a promising active packaging film was developed through a more environmentally friendly procedure and tested successfully for kiwi fruit preservation. As future work, we could say that this product should be also tested with other food kinds and develop an industrial process for bulk film production via a scale-up procedure.

## 4. Materials and Methods

### 4.1. Materials

Acros-Organics company was the supplier of Chitosan (CS) with a molecular weight of 100,000–300,000 (Zeel West Zone 2, Janssen Pharmaceuticalan 3a, B2440, Geel, Belgium). Poly(vinyl alcohol) (PVOH) (low molecular weight i.e., 13,000–23,000, hydrolysis degree 87–89%) was purchased by SIGMA-ALDRICH (Co., 3050 Spruce Street, St. Louis, MO, USA, 314-771-5765). Nanocor Inc. was provided the powder nanoclay (2870 Forbs Avenue, Hoffman Estates, IL, USA). Montmorillonite which contained 1.53% halloysite nanotubes (Al_2_Si_2_O_5_(OH)_4_∙2H_2_O, 99.5% clay, 1.68% CaO, 3.35% Fe_2_O_3_, 62.9% SiO_2_, 19.6% Al_2_O_3_, and 3.05% MgO) was obtained by Sigma-Aldrich (product 685445, Sigma-Aldrich, St. Louis, MO, USA). The used Thyme Oil (TO) was produced by the Chemco company (Via Achille Grandi, 13–13/A, 42030 Vezzanosul, Crostolo, Italy).

### 4.2. Preparation of TO@HNT Hybrid Nanostructure

**Figure 12:**
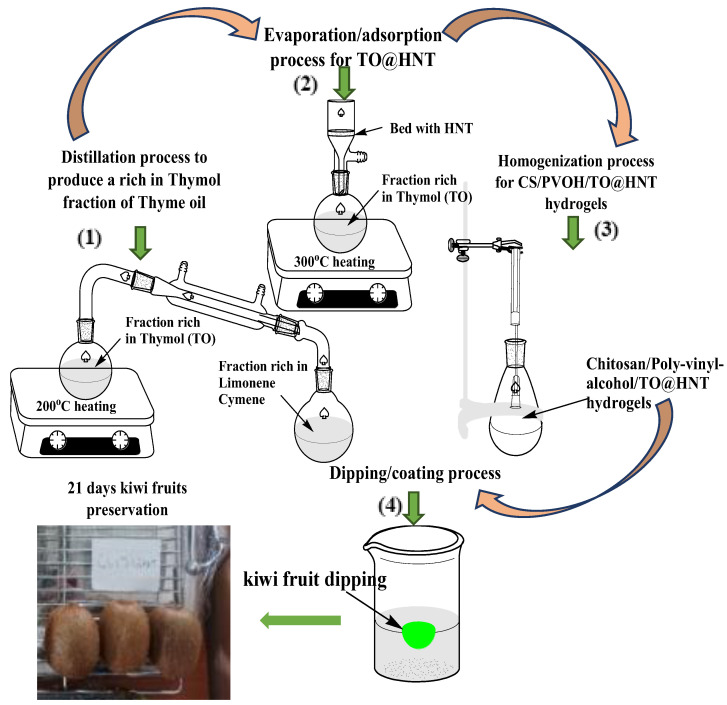
Schematic presentation of (**1**) distillation process to produce a rich in thymol fraction from pure thyme oil, (**2**) evaporation/adsorption process to modify pure HNT and develop TO@HNT nanohybrids, (**3**) homogenization process to develop CS/PVOH/TO@HNT gels and (**4**) kiwi fruit dipping/coating process.

The modification of the HNT nanoclay with TO EO was based on a modified evaporation/adsorption process [36]. Firstly, 20 mL of TO was placed in a glass distiller flask and heated at 200 °C to remove the D-limonene and L-cymene (see part (1) in Figure 12). The remaining rich in thymol TO was placed in a round bottom glass flask. Above the flask, 2 g of HNT bed was adapted and above the HNT bed, a reflux condenser was placed. With this modified reflux condenser, the remaining rich in thymol TO was heated at 300 °C and the evaporated thymol molecules adsorbed on HNT (see part (2) in Figure 12).

When the HNT bed color turned from white to brown the process stopped and the modified HNT was removed and weighted. The wt.%. TO loading to HNT was calculated at approx. 30%. The as-prepared rich in thymol-modified HNT was labeled as TO@HNT and used as active nano-reinforcement in the development of CS/PVOH/TO@HNT active films/coatings.

### 4.3. Preparation of CS/PVOH/HNT and CS/PVOH/TO@HNT Films/Coatings

For the preparation of each film/coating, 100 mL of an aqueous 2% *w*/*v* CS solution activated with 1% *v*/*v* acetic acid was used. In this 100 mL of as-prepared 2% *w*/*v* CS solution with 1% *v*/*v* acetic acid PVOH added to achieve final PVOH nominal content at 30% wt. The obtained mixture was refluxed overnight to solubilize PVOH and to achieve a homogeneous CS/PVOH solution. In this, 100 mL of CS/PVOH mixed solution amounts of TO@HNT hybrid nanostructure was added and homogenized for 5 min at 18,000 rpm to achieve final TO@HNT nominal content 5, 10, and 15 wt.% (see part (3) in Figure 12). For comparison amounts of pure HNT were also added into CS/PVOH mixed solution to achieve final HNT nominal content 5, 10, and 15 wt.%. The as-obtained CS/PVOH/xHNT and CS/PVOH/xTO@HNT (x takes values 5, 10, and 15) homogenized coatings were placed in 11 cm diameter petri dishes and dried at ambient temperature. The obtained films were peeled off and preserved inside PE plastic bags at 25 °C and 50% RH before the measurements.

### 4.4. Characterization of TO@HNT Hybrid Nanostructure

The obtained TO@HNT hybrid nanostructure was characterized with XRD analysis, FTIR spectroscopy, and DSC analysis. For XRD analysis a Brücker D8 advance instrument was employed (Brüker, Analytical Instruments, S.A., Athens, Greece) and the measurements were carried out in the range 2θ = 2–30°. For the FTIR spectroscopy measurements, an FT/IR-6000 JASCO Fourier transform spectrometer (JASCO, Interlab, S.A., Athens, Greece) was employed. The FTIR spectra were recorded in the range of 4000–400 cm^−1^ and the obtained spectra was was the average of 32 scans at 2 cm^−1^ resolution. For the DSC experiments, a DSC214 Polyma Differential Scanning Calorimeter (NETZSCH manufacturer, Selb, Germany) was employed. Samples with an average weight in the range of 1.2–3.3 mg were tested under a nitrogen atmosphere with a heating rate of 10 K/min from 50 to 300 °C.

### 4.5. XRD Analysis of CS/PVOH/HNT and CS/PVOH/TO@HNT Films

The prepared CS/PVOH/HNT and CS/PVOH/TO@HNT films were characterized with XRD analysis by using a Brücker D8 advance instrument was employed (Brüker, Analytical Instruments, S.A., Athens, Greece) in the range of 2θ = 2–30°.

### 4.6. FTIR Analysis of CS/PVOH/HNT and CS/PVOH/TO@HNT Films

The interaction of pure HNT with CS/PVOH matrix in CS/PVOH/HNT films and TO@HNT hybrid nanostructure with CS/PVOH matrix in CS/PVOH/TO@HNT films were tested with FTIR analysis by using an FT/IR-6000 JASCO Fourier transform spectrometer (JASCO, Interlab, S.A., Athens, Greece) in the range of 4000–400 cm^−1^.

### 4.7. SEM Images

The surface and cross-section morphology of CS/PVOH/HNT and CS/PVOH/TO@HNT films was recorded by using a JEOL JSM-6510 LV SEM (Microscope Ltd., Tokyo, Japan) were used equipped with an X-Act EDS-detector by Oxford Instruments, Abingdon, Oxfordshire, UK (an acceleration voltage of 20 kV was applied).

### 4.8. Tensile Properties of CS/PVOH/HNT and CS/PVOH/TO@HNT Films

The influence of pure HNT and TO@HNT addition in the tensile characteristics of the CS/PVOH matrix was evaluated with tensile measurements by using a Simantzü AX-G 5kNt instrument (Simandzu, Asteriadis, S.A., Athens, Greece). According to the ASTM D638 method, three to five samples of type V ASTMD638 specimens of each film were tensioned at an across-head speed of 2 mm/min.

### 4.9. UV-vis Transparency of CS/PVOH/HNT and CS/PVOH/TO@HNT Films

UV–vis transmittance measurements for all obtained CS/PVOH/HNT and CS/PVOH/TO@HNT films were carried out with a Shimatzu 1900 spectrophotometer in the range of 200 to 800 nm.

### 4.10. Water and Oxygen Barrier Properties of CS/PVOH/HNT and CS/PVOH/TO@HNT Films

For the evaluation of the water vapor transmission rate (WVTR) of CS/PVOH/HNT and CS/PVOH/TO@HNT films, a hand-made apparatus was used according to the ASTM E96/E 96M-05 method [66]. According to the methodology described extensively in previous reports [17,40,41,42,66] experiments were carried out inside thermostated chamber at 38 °C and constant 95% RH. Water vapor transmission rate WVTR (g∙cm^−2^∙s^−1^) and water vapor diffusion coefficient D (cm^2^∙s^−1^) were estimated according to Equations (2) and (3) respectively as they reported to number [50] referenced paper and based on the theory described in this paper [67].

### 4.11. Oxygen Permeability of CS/PVOH/HNT and CS/PVOH/TO@HNT Films

ASTM D 3985 method (23 °C and 0% RH) was followed to estimate the oxygen transmission rate (OTR). OTR values in cc O_2_∙m^−2^∙day^−1^ achieved using an (8001, Systech Illinois Instruments Co., Johnsburg, IL, USA) oxygen permeation analyzer. The oxygen permeability coefficient Pe_gas_ (cm^2^/s), was estimated following Equations (4) and (5) of the number [50] referenced paper and according to the theory described in the literature [68].

### 4.12. Antioxidant Activity of CS/PVOH/HNT and CS/PVOH/TO@HNT Films

The antioxidant activity of CS/PVOH/HNT and CS/PVOH/TO@HNT films were evaluated with the diphenyl-1-picrylhydrazyl (DPPH) method. Briefly, 300 mg of each film was cut into small pieces and placed inside dark glass bottles with 10 mL of an ethanolic solution of diphenyl-1-picrylhydrazyl (DPPH) with 40 ppm concentration. A sample with 10 mL of ethanolic DPPH solution without the addition of any film was used as the blank sample. The absorbance of the DPPH solution at 517 nm at 0 h and after 24 h incubation was measured using a Jasco V-530 UV-vis spectrophotometer. For each film, three to five different samples were made and measured. After 24 h incubation of films, the % antioxidant activity was estimated using the Equation (7) reported in previous reports [27,40,41,49].

### 4.13. Antibacterial Activity of CS/PVOH/HNT and CS/PVOH/TO@HNT Films

Antibacterial properties of the films were investigated by utilizing the agar diffusion method against Gram-negative bacteria *Escherichia coli* (ATCC 25922), *Salmonella enterica* subsp. enterica (DSMZ 17420), Gram-positive bacteria *Staphylococcus aureus* (DSMZ 12463), and *Listeria monocytogenes* (DSMZ 27575). The tested foodborne microorganisms were obtained from the Institute of Technology of Agricultural Products, ELGO-DEMETER, Lykovryssi, Greece.

Fresh cultures of the bacterial strains were prepared in Mueller Hinton Broth. The cultures were inoculated at 37 °C for 24 h in order to achieve a range of 10^7^–10^8^ CFU mL^−1^. After that, the bacteria were swabbed on Mueller-Hinton agar dishes by rotating the plate every 60° to ensure consistent growth.

The tested films were cut into 6 mm diameter discs by a circular knife and were placed on a Mueller-Hinton inoculated plate. The dishes were incubated at 37 °C overnight. The diameter of the inhibitory zones, and the contact area of the discs with agar surface, were measured. The experiment was performed thrice.

### 4.14. Packaging Test of CS/PVOH/HNT and CS/PVOH/TO@HNT Coatings in Preservation of Kiwifruits

15 kiwifruits of as close to the same shape as possible and the same ripeness were purchased from the local supermarket and they were divided into four groups with three fruits each. The first group of three kiwifruits was used as blank uncoated samples. The other three groups with three kiwifruits each were coated with pure CS/PVOH solution, CS/PVOH/10HNT solution, and CS/PVOH/10TO@HNT. CS/PVOH/10TO@HNT coating solution was selected as the optimum one according to each higher water/oxygen barrier properties shown hereabove which are critical for such active fruit coatings. Therefore, the uncoated and coated kiwifruits were put in a tray, stored under room humidity at 25 °C, and observed daily for any visible changes or fungal growth on their surfaces over 21 days. For the coating of kiwi fruits, dipping for 1 min in the selected coating solutions was followed (see part (4) in Figure 12).

### 4.15. Statistical Analysis

Three pieces of every film sample were tested to obtain the values presented in Table 1, Table 2, Table 3 and Table 4. The final value of each property is the mean value of such measurements. All experimental data were processed with the SPSS vr. 20 statistical software and the mean values and standard deviation values which are tabulated above, resulted assumed a confidence interval of C.I. = 95%. Hypothesis tests ran assuming a statistical significance level of *p* = 0.05 to ensure that different mean values of a property for different samples are also statistically different. The non-positive normality tests implied the non-parametric Kruskal–Wallis method for such investigations and statistically unequal mean values of all properties were confirmed. The equality or inequality assurance was tested according to the empirical Equations (1) and (2) which were explained in detail in previous works [40]:

(1) $$\mathrm{EA}\left(\%\right)=\frac{\mathrm{Sig}.-p}{1-p}\cdot 100$$

(2) $$IA\left(\%\right)=\frac{p-\mathrm{Sig}.}{p}\cdot 100$$

All the mechanical and barrier properties of all kinds of films exhibit statistical inequal mean values. The significance Sig. value range resulting from the SPSS software is presented in the following table:

It is obvious from Table 5 that the values of properties E, σ_uts_, ε%, WVTR, and OTR are different for different kinds of films while the values of the antioxidant activity are different but, in some cases, close to each other. Finally, it is obvious from Table 4 and Table 5 that mean values of antimicrobial activity, in some cases are statistically equal while in other cases are statistically unequal.

## Figures and Tables

**Table 1:** Calculated values of Young’s (E) Modulus, ultimate tensile strength (σuts), and % strain at break (ε_b_).

Sample Name	E-Elastic Modulus (MPa)	σ _uts_ (MPa)	ε%-Elongation at Break
CS/PVOH	2249.3 ± 20.0	71.2 ± 1.8	14.8 ± 0.9
CS/PVOH/5HNT	2552.0 ± 21.3	74.1 ± 2.1	7.0 ± 1.6
CS/PVOH/10HNT	2766.0 ± 35.1	79.8 ± 1.7	5.7 ± 0.8
CS/PVOH/15HNT	2865.0 ± 27.4	96.0 ± 1.2	6.7 ± 0.6
CS/PVOH/5TO@HNT	2644.5 ± 13.4	74.9 ± 2.3	7.2 ± 2.1
CS/PVOH/10TO@HNT	2993.7 ± 27.6	103.7 ± 1.4	7.1 ± 0.2
CS/PVOH/15TO@HNT	2965.0 ± 29.4	98.5 ± 1.5	6.8 ± 0.7

**Table 2:** Film thickness, water vapor transmission rate (WVTR), water diffusivity (D), oxygen transmission rate (OTR), and oxygen diffusivity (Pe_O2_) values of pure CS/PVOH film as well as CS/PVOH/HNT and CS/PVOH/TO@HNT films.

Sample Name	Film Thickness (mm)	WVTR × 10^−6^ (gr × cm^−2^ × s^−1^)	D × 10^−4^ (cm^2^ × s^−1^)	OTR (mL × m^−2^ × day^−1^)	Pe_O2_ × 10^−7^ (cm^2^ × s^−1^)
CS/PVOH	0.140 ± 0.010	1.15 ± 0.13	3.65 ± 0.31	49,577 ± 2478	8.03 ± 0.40
CS/PVOH/5HNT	0.113 *±* 0.025	1.42 ± 0.18	3.67 ± 0.72	57,345 ± 2867	7.52 ± 0.38
CS/PVOH/10HNT	0.123 ± 0.015	1.02 ± 0.13	2.83 ± 0.62	32,785 ± 1639	4.68 ± 0.23
CS/PVOH/15HNT	0.117 ± 0.012	1.03 ± 0.20	2.75 ± 0.17	44,234 ± 2211	5.97 ± 0.30
CS/PVOH/5TO@HNT	0.140 ± 0.010	1.14 ± 0.13	3.59 ± 0.14	43,345 ± 2167	7.02 ± 0.35
CS/PVOH/10TO@HNT	0.117 ± 0.021	1.02 ± 0.21	2.62 ± 0.17	28,974 ± 1449	3.91 ± 0.20
CS/PVOH/15TO@HNT	0.170 ± 0.017	1.01 ± 0.67	3.64 ± 0.14	30,434 ± 1521	5.64 ± 0.28

**Table 3:** Antioxidant activity values of pure CS/PVOH and all CS/PVOH/HNT, CS/PVOH/TO@HNT films.

Sample Name	Antioxidant Activity after 24 h ^1^ (%)
CS/PVOH	3.5 ± 1.6
CS/PVOH/5HNT	5.4 ± 2.6
CS/PVOH/10HNT	5.5 ± 2.2
CS/PVOH/15HNT	6.1 ± 3.1
CS/PVOH/5TO@HNT	18.1 ± 5.0
CS/PVOH/10TO@HNT	25.5 ± 3.6
CS/PVOH/15TO@HNT	32.2 ± 5.1

^1^ DPPH assey.

**Table 4:** Antibacterial activity of active films against food pathogenic bacteria *E. coli*, *S. enterica*, *S. aureus*, and *L. monocytogenes*.

Film Material	*E. coli*		*S. enterica*		*S. aureus*		*L. monocytogenes*	
	Inhibition ^a^	Contact ^b^	Inhibition ^a^	Contact ^b^	Inhibition ^a^	Contact ^b^	Inhibition ^a^	Contact ^b^
CS	0.00	-	0.00	-	0.00	-	0.00 ^9^	+
CS/20PVOH	3.50 ± 0.87	-	3.83 ± 0.76 ^5,6,8^	-	4.47 ± 0.81 ^3^	-	0.00 ^9^	+
CS/20PVOH/5HNT	4.83 ± 0.29 ^1^	-	5.00 ± 0.00 ^5,6,7,8^	-	5.40 ± 0.66 ^3,4^	-	5.00 ± 0.00 ^10^	-
CS/20PVOH/10HNT	5.00 ± 0.00 ^1^	-	5.33 ± 0.76 ^6,7,8^	-	5.77 ± 0.75 ^3,4^	-	5.00 ± 0.00 ^10^	-
CS/20PVOH/15HNT	7.00 ± 0.50 ^2^	-	6.33 ± 0.29 ^6,7,8^	-	6.00 ± 0.00 ^4^	-	6.57 ± 0.51	-
CS/20PVOH/5TO@HNT	7.50 ± 0.50 ^2^	-	6.83 ± 0.29^8^	-	9.00± 0.00	-	8.00 ± 0.50	-
CS/20PVOH/10TO@HNT	7.80 ± 0.20	-	8.00 ± 0.00	-	9.50 ± 0.50	-	9.03 ± 0.45	-
CS/20PVOH/15TO@HNT	8.00 ± 0.50	-	9.00 ± 0.87	-	10.00 ± 0.00	-	9.00 ± 0.50	-

^a^ Inhibitory zone surrounding film discs measured in mm after the subtraction of the disc diameter (6 mm); ^b^ Contact area of film discs with the agar surface; (+) indicates bacterial growth in the area, (-) indicates no bacterial growth in the area; Results expressed as mean ± standard deviation (*n* = 3); Means in the same column baring same superscript numbers are significantly equal (*p* > 0.05).

**Table 5:** Significance level, equality, and inequality, assurance of mean values for Young Modulus (E), σ_uts_, % elongation at break (ε%), WVTR, OTR, % Antioxidant.

	E	σ_uts_	ε%	WVTR	OTR	Antiox.	*E. coli*	*Saur.*	*Senter.*	*L. monoc.*
Sig. < 0.05	0–0.0110	0–0.0185	0–0.014	0–0.0220	0–0.0120	0–0.0400	0–0.037	0–0.024	0–0.039	0–0.002
IA (%)	78–100	63–100	72–100	56–100	76–100	20–100	26–100	52–100	22–100	96–100
Sig. > 0.05	-	-	-	-	-	-	0.862–1	0.071–0.999	0.081–0.991	1
EA (%)	-	-	-	-	-	-	85–100	2–100	3–99	100

Significance level *p* < 0.05.

## Data Availability

The datasets generated for this study are available on request to the corresponding author.
